# Event and Comment

**Published:** 1922-08

**Authors:** 


### DENTAL REGISTER

THE MAGAZINE OF DENTISTRY

Vol. LXXVI AUGUST, 1922 No. 8

### EVENT AND COMMENT

**Recreation Requirements.** By force of habit, or because of custom or tradition, we seem to have acquired periods of vacation when we believe it is desirable that we abstain from the routines and the continuous endeavors we make to keep the machinery of life in a more or less perpetual state of activity. At times it even seems necessary that the natural periods of sleep have to be curtailed, and sometimes profound sleep passes in a semi-automatic state of feverish unrest which lacks refreshing and vitalizing influences. In the end such living and recreation results in loss of vitality and we die before the natural time from sheer fatigue or exhaustion. The cause for this climacteric state of untimely death is due to our practice of fast and strenuous living to meet the needs of men's ambitions and it may, in great measure, also be due to the fact that impaired health causes nervous breakdowns from inordinate eating and drinking of good food improperly utilized. Many chronic diseases are traceable to bad eating. All these incidents are more evident than formerly because people are not satisfied to live the simple lives our fathers were forced to adopt.

In spite of the fact that medical science has taught us how to take better care of ourselves the span of life has not materially lengthened, because there has, during this period, come about a greater demand for a more intricate scheme of living which is both exacting and exhaustive.

It can be likened to the modern high-power machinery with such a rate that the enormous friction wears it out quicker than usual.

We are wearing ourselves out in an untimely way, because we are unwilling to take a moderate gate, and don't use sufficient and proper lubrication. When we expostulate with such men, even when in the prime of life and manhood, they will naively say to you, that this is the only way one can hope to get on and get fame or fortune. Instead of slacking their pace they open wider the throttle and hope by good management and some happy chance to make their goal safely, but too often the increased speed and the faulty machine lands us in the ditch. The worst feature of this situation is that we have failed to supply adequate safeguards. Our medical confreres understand what is going on but they seem to be wholly occupied, like the dentists, in taking care of the wreckage. They know that the skill of the surgeons and internists will do all that is practicable when the break comes, but they can not be responsible for other's recklessness.

As a matter of fact what is the dental profession doing more than usual to stabilize the health of the dental practitioners? With our constantly growing technical and professional knowledge are we really making a proper effort to make ourselves more fit to withstand the ever increasing strain on modern dental practice; or can we expect the practitioner of tomorrow will be able to carry on the larger duties that seem imminent, unless he can learn to conserve his own resources better than he has done heretofore? We have no statistics upon which to base an opinion of this matter but from general observations we don't believe dentists are taking adequate care of their own health as they should. At any rate we believe that it is high time most of us were inspecting our brakes and looking out for ourselves.

It seems almost impracticable for most dentists to take a serious overlooking of their own conditions of health. They don't even put their own mouths in the hands of the dental hygienists. They never take a day off for healthful recreation, as though it would be sinful, or a waste of time and money, as a matter of fact too many dentists are such poor business men that they can not really loose even an hour's time; of course there is no hope for such a one.

It does seem that every dentist could find time for regular and helpful exercise and diversion from the usual tedious and tiresome days of grind and endeavor; in fact this should not only be desirable but it should be mandatory. The form and character of rest can't be determined for every one, but it should be ample and sufficient. Change of occupation, out of door exercises, and rest from eye-strain, and all long-continued positions, are highly essential and helpful.

The first and most important duty of the dentist is that he study his profession and acquaint himself with its demands that he may cultivate a sincere love for his task. This will create a capacity for good work and give him a wholesome tire of body that will force him to think of himself as well as his patients in a healthy and wholesome way. The dentist should keep well so he can serve adequately, and he should have a bright and helpful disposition that will minister to his own health and happiness.

This *Comment* is inspired by a contribution which the editor of the *Dental Digest* makes on the subject of the dentist's health. He writes feelingly as well as intelligently, because he has experienced the ill effects of excessive work and lack of recreation.

